# Development of Thin‐Film Composite Membranes from Aromatic Cardo‐Type Co‐Polyimide for Mixed and Sour Gas Separations from Natural Gas

**DOI:** 10.1002/gch2.201900107

**Published:** 2020-03-12

**Authors:** Garba O. Yahaya, Seung‐Hak Choi, Melhan M. Ben Sultan, Ali Hayek

**Affiliations:** ^1^ Research & Development Center Saudi Aramco P.O. Box 62 Dhahran 31311 Saudi Arabia

**Keywords:** natural gas, permeability, polyimides, thin films

## Abstract

The consumption of natural gas (NG) is rapidly increasing worldwide as it is becoming the second largest fuel source after coal. However, many of the world gas reserves contain high levels of subquality NG including the presence of carbon dioxide (CO_2_), hydrogen sulfide (H_2_S), nitrogen (N_2_), benzene/toluene/xylene (BTX) etc., in varying amounts (up to 50% v/v in some reserves), which constitute several problems. Membrane‐based NG sweetening/upgrading processes emerge as among the fastest growing technologies, due to their lower capital cost, higher energy savings, greater economic viability, etc. as compared to conventional technologies. Thus, a defective‐free, multilayer thin‐film composite membrane is developed from 6FDA‐Durene/6FDA‐CARDO block co‐polyimide for the separation of sour gas from NG. The membrane shows good performance as it exhibit CO_2_/CH_4_ and H_2_S/CH_4_ selectivities ranges from 8 to 10 and 15 to 19, respectively, and CO_2_ and H_2_S permeance are 122 and 220 GPU, respectively.

## Graphical abstract

A defective‐free multilayer thin film composite membrane is developed from block co‐polyimide for the separation of sour gas from natural gas. The thin‐film composite membrane shows good performance under high H_2_S environment. The membrane exhibits CO_2_/CH_4_ and H_2_S/CH_4_ selectivities ranges from 8 to 10 and 15 to 19, respectively, and CO_2_ and H_2_S permeances are up to 122 and 220 GPU, respectively.

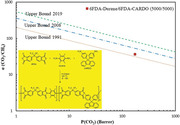

## 1 Introduction

In the last several years, gas separation membranes have found industrial applications and have been very attractive to several gas separation processes including, natural gas sweetening, and upgrading.^[^ ^1^ ^]^ This is due to its high energy efficiency, small footprint, and lower cost than the traditional techniques that include absorption, adsorption, and cryogenic distillation.^[^ ^1^, ^2^ ^]^ In view of this and the fact that membrane gas separation has found a few applications in industry so far (i.e., large‐scale production and purification of methane and CO_2_ from biogas),^[^ ^3^ ^]^ tremendous studies in the field are ongoing and novel materials are being developed^[^ ^4^, ^5^, ^6^, ^7^, ^8^, ^9^, ^10^, ^11^, ^12^, ^13^, ^14^, ^15^, ^16^, ^17^, ^18^, ^19^, ^20^, ^21^, ^22^, ^23^, ^24^, ^25^, ^26^, ^27^, ^28^, ^29^, ^30^, ^31^, ^32^, ^33^, ^34^, ^35^, ^36^ ^]^ in order to improve membrane performance and address the challenges currently being encountered in gas separation membrane applications.

The consumption of natural gas (NG) is rapidly increasing worldwide, as it is becoming the next or second largest fuel source after coal.^[^ ^15^ ^]^ However, many of the world gas reserves contain high levels of sub‐quality natural gas including presence of CO_2_, H_2_S, N_2_, and heavy hydrocarbon, in varying amounts (up to 50% v/v in some reserves). These contaminants could constitute major problems including pipeline corrosion, decrease in heating value, and upsurge in the cost of compression and transportation if not separated from NG streams. In addition, H_2_S is highly toxic and its combustion leads to harmful sulfur dioxide and thus has to be removed from the NG streams in order to meet sales gas specification and mitigate all the problems discussed earlier.^[^ ^16^ ^]^

Membrane gas separation and its hybrid with absorption processes are gaining tremendous attention in research due to their remarkable benefits discussed earlier.^[^ ^17^, ^18^, ^19^, ^20^, ^21^ ^]^ However, the existing membranes performances are much below expectation and this hampers membrane application opportunities on large industrial scale.^[^ ^22^, ^23^ ^]^ The difficulty in achieving simultaneous high permeability and high selectivity (trade‐off), plasticization, and the aging of the membrane are some of the challenges that have to be addressed. For instance, membrane plasticization significantly degrades the permeation properties of membrane at high gas pressures and in the presence of other components such as BTX, heavy hydrocarbons, water. In addition, membrane physical aging reduces the permeability of membrane considerably over the membrane average lifespan (i.e., 2–5 years). These are impediments to long‐term stability and membrane performance. In view of this, membranes with higher performance are very crucial to the economic viability of its applications in various gas separation processes. Furthermore, high membrane performance is very vital and important in order to be competitive with conventional separation methods that include pressure swing adsorption, amine absorption process, and cryogenic distillation.^[^ ^18^, ^19^, ^20^, ^21^, ^22^, ^23^ ^]^ Thus, in order to mitigate all these challenges, several research efforts are ongoing to develop high‐performance gas separation membranes.^[^ ^4^, ^5^, ^6^, ^7^, ^8^, ^9^, ^10^, ^11^, ^12^, ^13^, ^14^, ^15^, ^16^, ^17^, ^18^, ^19^, ^20^, ^21^, ^22^, ^23^, ^24^, ^25^, ^26^, ^27^, ^28^, ^29^, ^30^, ^31^, ^32^, ^33^, ^34^, ^35^, ^36^ ^]^

A lot of attention has been concentrated on aromatic polyimides in the last few years in view of their outstanding properties that include excellent resistance to chemical, thermal, and mechanical as well as excellent transport properties, especially very high selectivities for gas pairs that include He/CH_4_, O_2_/N_2_, H_2_/CH_4_, and CO_2_/CH_4_.^[^ ^23^, ^24^, ^25^, ^26^, ^27^, ^28^, ^29^, ^30^, ^31^, ^32^, ^33^, ^34^, ^35^, ^36^ ^]^ The fluorinated polyimide exhibits high CO_2_ permeability as well.^[^ ^25^, ^26^ ^]^ 6FDA‐based polyimide is one type of aromatic polyimide that has received a lot of interest for separation of gas in the last few decades. This is due to their high glass transition temperature and other excellent properties previously discussed for polyimide family. These attributes certainly facilitate their application in especially aggressive environments, such as the NG sweetening process.

Despite the fact that some of the 6FDA‐based polyimides show high performance for several gas pairs^[^ ^23^, ^24^, ^25^, ^26^ ^]^ and have exhibited potential opportunities for more basic research, they are far from being applied in industrial scale. In general, vast majority of data obtained from all the studies earlier described^[^ ^4^, ^5^, ^6^, ^7^, ^8^, ^9^, ^10^, ^11^, ^12^, ^13^, ^14^, ^15^, ^16^, ^17^, ^18^, ^19^, ^20^, ^21^, ^22^, ^23^, ^24^, ^25^, ^26^, ^27^, ^28^, ^29^, ^30^, ^31^, ^32^, ^33^, ^34^, ^35^, ^36^ ^]^ were pure gas tested on a dense membrane with a membrane thickness of up to 150 µm or more. However, these data do not represent and vary greatly from the industrial value data. This is due to the fact that the transport behavior of thin film composite (TFC) membranes with a thin selective layer of about 0.2–1.0 µm thick are usually different from those of the self‐supporting dense membrane. This may be due to the different polymer chain arrangements in TFC and dense films,^[^ ^1^, ^2^, ^3^, ^4^ ^]^ which then result in different permeation properties and separation performances.^[^ ^12^ ^]^

One of the most challenging issues being encountered in many recent studies is the expensive fabrication of the 6FDA‐based polyimide as solely integral asymmetric hollow fiber membranes.^[^ ^37^, ^38^, ^39^, ^40^, ^41^, ^42^ ^]^ The other challenging issue is the fabrication of a defective‐free ultrathin multilayer with 6FDA‐based polyimide as a selective layer without losing separation performance for industrial scale separation processes.^[^ ^42^ ^]^ Even though hollow fiber membranes have greater benefits as compared to flat‐sheet membranes, such as higher packing density, higher area per module, much easier to scale up, lower manufacturing cost,^[^ ^4^, ^8^, ^10^ ^]^ however, only few studies have been reported on the fabrication of 6FDA‐based polyimide thin film composite hollow fiber membranes.^[^ ^42^, ^43^, ^44^, ^45^, ^46^, ^47^ ^]^ Moreover, about 50 g m^−2^ of polymer is required for the fabrication of hollow fiber membranes using phase inversion techniques.^[^ ^48^ ^]^ Thus, it may not be economically viable to use this method to fabricate membranes for especially very expensive polymers.^[^ ^48^ ^]^ However, thin film composite membranes have more advantages when compared to asymmetric hollow fiber membranes. The fabrication of TFC requires less limitations on the mechanical and process of the materials, and a much smaller amount (0.1–2 g m^−2^) is required to be deposited on the substrate of a composite membrane; thus, very costly materials with very high performance can be used.^[^ ^48^ ^]^

In view of the numerous benefits of multilayer composite membranes as previously discussed, the concept can be applied to fabricate 6FDA‐based polyimide based composite membranes. The TFC membranes prepared using the solution coating method have received a lot of attention in the last few years and many high performance and useful membranes have been fabricated^[^ ^11^, ^42^, ^43^, ^44^, ^45^, ^46^, ^47^, ^48^, ^49^, ^50^, ^51^, ^52^, ^53^ ^]^ using this method. Moreover, very high gas permeance is obtained when the selective layer thickness is very small (less than 1.0 µm).^[^ ^1^ ^]^ Such membrane development has been the focus of many studies^[^ ^42^, ^43^, ^44^, ^45^, ^46^, ^47^, ^48^, ^49^, ^50^, ^51^, ^52^, ^53^, ^54^ ^]^ in the last few years. Therefore, this study is focused on development and fabrication of 6FDA‐based thin films composite membranes and investigation of their pure, mixed, and sour gas permeation properties under multiple operating conditions. The block co‐polyimide with a 6FDA‐Durene/6FDA‐CARDO (5000:5000) backbone was employed as the selective layer. This was recently developed from the co‐polymerization of 6FDA‐Durene and 6FDA‐CARDO.^[^ ^26^ ^]^

## 2 Experimental Section

### 2.1 Materials

The block co‐polyimides were synthesized in the previous study,^[^ ^26^ ^]^ using 6FDA (Alfa Aesar, Haverhill, MA, USA), Durene diamine (Tokyo Chemical Industry, Tokyo, Japan), and CARDO (Aldrich, St. Louis, MO, USA). The monomers were used as received without further purification. PTMSP (Gelest Inc., Morrisville, PA, USA) (Fisher Chemical, Hampton, NH, USA) was used as received to form a gutter layer on top of mesoporous polyacrylonitrile substrate (PAN350, MWCO = 20 kDa, Sepro Membrane, Carlsbad, CA, USA). Cyclohexane (99.5%, Sigma‐Aldrich, St. Louis, MO, USA), chloroform (≥99.8%, Sigma‐Aldrich, St. Louis, MO, USA), *n*‐hexane (97%, Sigma‐Aldrich, St. Louis, MO, USA), *m*‐cresol (Alfa Aesar, Haverhill, MA, USA), and methanol (ThermoFisher Scientific, Waltham, MA, USA) were used as solvents, as received.

### 2.2 Synthesis and Characterization of Block Co‐Polyimide Containing 6FDA‐Durene/6FDA‐CARDO (5000/5000) Backbone

**Figure 1:**
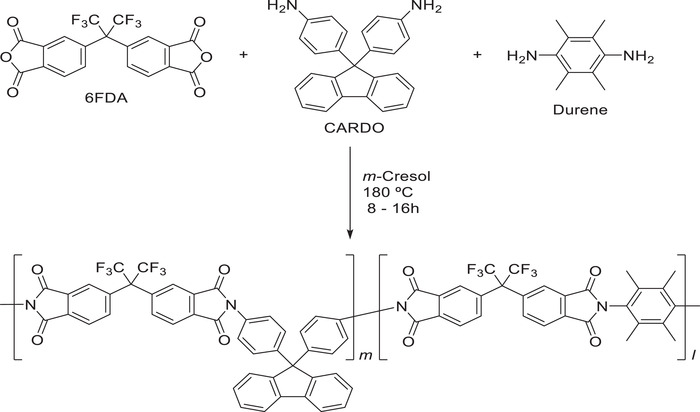
Synthetic scheme of block co‐polyimide 6FDA‐Durene/6FDA‐CARDO (5000/5000).

Block co‐polyimides containing 6FDA‐Durene/6FDA‐CARDO (5000/5000) backbone (**Figure** **1**) were synthesized and characterized in the previous publication.^[^ ^26^ ^]^ Monomer compositions used in the synthesis are depicted in **Table** **1**.

**Table 1:** Monomers composition in the synthesized co‐polyimide

Co‐polyimide	m_(CARDO)_ [mmol]	m_1(6FDA)_ [mmol]	m_(Durene)_ [mmol]	m_2(6FDA)_ [mmol]
6FDA‐Durene/6FDA‐CARDO (5000/5000	15	12.74	16.83	19.09

### 2.3 Membrane Preparation

#### 2.3.1 Thin Film Composite Block Co‐Polyimide Membrane

The thin film composite membranes were developed using a spin coater (KW‐4A, Chemat Technology, Northridge, CA, USA) and detailed procedure has been described in a previous publication.^[^ ^43^ ^]^ The PTMSP gutter layer was firstly formed on a PAN 350 UF membrane using a spin coater. Basically, the coating and caulking procedures were performed by first cleaning the PAN 350 UF membrane and then positioning it on a vacuum chuck (rotating disk) with a tape to form a flat surface. The spin coater's speed was set to a target speed and 1 mL of PTMSP gutter layer solution (2 wt% of PTMSP dissolved in cyclohexane) was then dripped dropwise onto the UF support for a predetermined coating/caulking time. The membrane was then placed in an oven to dry at about at 80 °C overnight. Then 4 wt% of 6FDA‐CARDO/6FDA‐Durene (5000/5000) co‐polyimide^[^ ^26^ ^]^ was dissolved in chloroform and filtered before used. The same procedure was then followed to coat the co‐polyimide selective layer on top of the PTMSP‐guttered PAN support. The coating thickness of gutter layer and selective layer was controlled by changing the polymer contents in the coating solution and coating speed and time. The co‐polyimide coated multilayer composite membrane was dried in an oven at 60 °C overnight. The membrane coating thickness was obtained from gas flux measurements using Equation (4) and confirmed by the scanning electron microscopy (SEM) micrograph cross section. The coating conditions were set to obtain 1–3 µm of the selective layer.

### 2.4 Gas Permeation and Membrane Characterization Measurements

The morphologies of the multilayer composite membranes were characterized by scanning electron microscopy (FEI QANTA 400F E‐SEM, ThermoFisher Scientific, Waltham, MA, USA), as described in a previous publication.^[^ ^43^ ^]^

**Figure 2:**
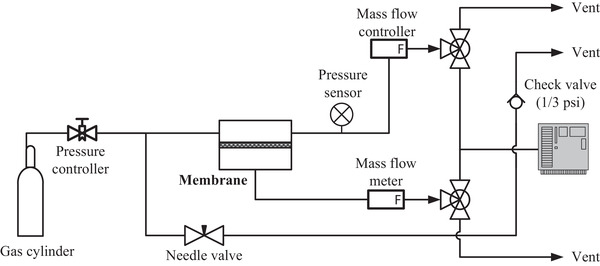
Schematic diagram of constant pressure system used for measuring pure and mixed gas permeation properties.

The sweet mixed gas transport properties of the composite membranes were analyzed for N_2_, CH_4_, C_2_H_6_, and CO_2_ gases at a feed pressure of up to 55 bar using a constant pressure system, as shown in **Figure** **2**, and well described in detailed in a previous publication.^[^ ^43^ ^]^ The gas permeation process can be described using CO_2_ as an example of feed gas as follows: The feed pressure of CO_2_ in the cylinder was regulated to a specific pressure using a pressure controller, as shown in Figure 2. Then the CO_2_ is passed through the retentate side of the membrane while its pressure was being monitored by the pressure sensor. This is required to monitor the occurrence of any pressure drop between the cylinder and retentate side of the membrane. For a certain stage‐cut, the flowrate of the CO_2_ in the retentate was controlled using a mass flow controller before venting it. Then part of the CO_2_ in the retentate permeated across the membrane and permeate flowrate was measured by the flowmeter in the permeate before venting it. The permeate was maintained at atmospheric pressure. For mixed gas permeation experiments, the needle and check valves as shown in Figure 2 were used to bypass the feed gas to the gas chromatography (GC) to measure feed gas composition and the GC was also used to measure the permeate gas composition, as shown in Figure 2.

The sour gas mixture permeation properties were also measured at 25 °C and feed pressure of up to 48 bar using a sour gas mixture containing 10, 10, 59, 1.0, and 20 vol% of H_2_S, CO_2_, CH_4_, C_2_H_6_, and N_2_, and respectively. For each experimental condition, three membranes were tested and the permeation test on each of these membranes were repeated three times to guarantee the absence of any deficiency and to ensure measurements are reproducible. The accuracy of the measurements were less than ±5% of the value shown

The two main factors to assess the permeation properties of membranes for gas separation are gas permeability, *P_i_*, which is also known as the permeability coefficient, and selectivity (α_*ij*_). These can be expressed as:^[^ ^55^ ^]^

(1) $$P_{i}=D_{i}\;\;S_{i}=\frac{j_{i}\;.l}{p_{i\left(0\right)}-p_{i\left(l\right)}}\;$$

where *j_i_*: flux of a species *i* (cm^3^(STP) cm^‐2^·s); *l*: thickness of membrane (cm); *p_i_*_(o)_: partial pressure of species *i* at the feed side (cmHg); *p_i_*_(*l*)_: partial pressure of species *i* at permeate side (cmHg); *D_i_*: diffusion coefficient of species *i* in the membrane [cm^2^ s^‐1^]; *S_i_*: solubility coefficient (cm^3^ (STP) of penetrant gas cm^‐3^ of polymer per pressure); *P_i_*: gas permeability of component *i* (barrer). (*D*_*i*_ *S_i_*) is referred to as the permeability of gas species *i*, (*P_i_*), and it is a product of diffusion and solubility coefficients. The permeability unit is barrer, where 1 barrer = 10^−10^ (cm^3^_STP_ cm)/(cm^2^ s cmHg).

Ideal selectivity (α_*ij*_) can be expressed as the membrane permeability ratio of two single gases as depicted in Equation (2):

(2) $$\alpha_{ij}=\frac{P_{i}}{P_{j}}\;$$

Permeability coefficients of each gas species in the mixed gas at low pressure can be calculated from Equation (3):

(3) $$\;P_{i}=\frac{x_{i\left(l\right)}J_{i\;}.l}{\left(P_{f}x_{i\left(0\right)}-P_{p}x_{i\left(l\right)\;}\right)}\;$$

where *x_i_*_(0)_ & *x_j_*_(0)_: gas mole fractions of components in the feed; *x_i_*(*l*) & *x_j_*(*l*): gas mole fractions of components in the permeate; *p_f_*: gas pressures (cmHg absolute) on the feed side; pp: gas pressures (cmHg absolute) on the permeate side.

Permeance (*Qi*) is mostly used to evaluate membrane performance for composite membranes, and thus permeance can be expressed as Equation (4):

(4) $$\;Q_{i}=\frac{P_{i}}{l}\;$$

The widely used unit for gas permeance is GPU (gas permeation unit), which is given as 1 GPU = 10^−6^ cm^3^_STP_/(cm^2^ s cmHg).

The separation factor can be expressed as the ratio of the composition of the feed gas to the permeate gas, which is given as Equation (5):

(5) $$\;\alpha_{i/j}^{m}=\frac{x_{i\left(l\right)}\text{/}x_{j\left(l\right)}}{x_{i\left(0\right)}\text{/}x_{j\left(0\right)}}\;$$

For composite membranes, the separation factor is usually used instead of selectivity, especially in mixed gas measurements. However for non‐ideal gas mixtures, selectivity $\propto_{i\text{/}j}^{m,*}$ is used,^[^ ^17^, ^27^, ^28^ ^]^ and this is expressed as

(6) $$\;\propto_{i/j\;}^{m,*}=\frac{P_{i}^{*}}{P_{j}^{*}}\;$$

where $P_{i}^{*}$: gas mixture fugacity‐based permeabilities of species *i* (barrer); $P_{j}^{*}$: gas mixture fugacity‐based permeabilities of species j (barrer).

As can be seen in Equations (1) and (3), effective membrane thickness in composite or asymmetric membranes is very important, however this cannot be measured easily. Thus, permeance *Qi*, as shown in Equation (4), is used to assess the permeation properties in the TFC rather than using permeability. The influence of the gas feed pressure on the permeance and permeate CO_2_ purity were studied. All the measurements were conducted at around 25 °C, feed pressure of up to 55 bar and stage‐cut of up to 20%. The permeate flowrates obtained vary from 12 to 9 cm^3^ s^−1^ for a stage‐cut of 5% to 20%, respectively. The permeate pressure was maintained at 0.07–0.21 bar. Detailed calculation methods for the stage‐cut and CO_2_ recovery is well described in a previous publication.^[^ ^43^ ^]^

## 3 Results and Discussion

### 3.1 Thin Film Composite Membrane Morphology

**Figure 3:**
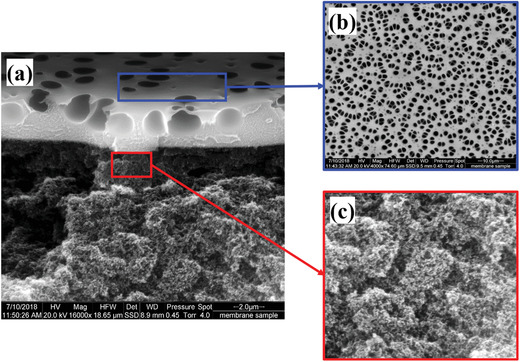
SEM images of a) cross‐section, b) surface, and c) enlarged (30000 magnification) cross‐sectional view of interface between substrate and coating layer of the multilayer thin film 6FDA‐Durene/6FDA‐CARDO (5000/5000) block co‐polyimide membrane.

As depicted in **Figure** **3**, a thin layer of co‐polyimide selective layer was successfully coated on mesoporous PAN substrate. However, it is interesting to observe that some pores were formed underneath of the selective layer, and approximately 2.0–2.5 µm of dense co‐polyimide selective layer was observed. Even though this may look porous based on the cross‐sectional image (Figure 3a), the pores do exist only up to very small thickness of co‐polyimide layer. It is believed that the pores were formed as a result of the rapid solvent (chloroform) evaporation that was used to dissolve the co‐polyimide. Due to the dramatic increase of polymer concentration by fast evaporation of the solvent, the pores cannot be re‐sealed.

### 3.2 Single Gas Permeation Properties of Dense Block Co‐Polyimide Membrane

**Figure 4:**
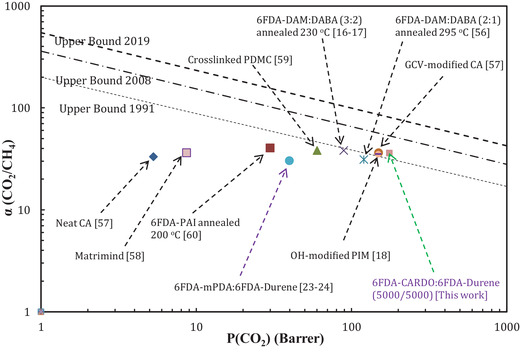
Comparison of pure gas CO_2_/CH_4_ permeability–selectivity trade‐off curve of the block co‐polyimide 6FDA‐Durene/6FDA‐CARDO (5000/5000) membrane and other membranes reported in the literature.^[^ ^16^, ^17^, ^18^, ^23^, ^24^, ^56^, ^57^, ^58^, ^59^, ^60^ ^]^

The block co‐polyimides containing a 6FDA‐Durene/6FDA‐CARDO (5000:5000) backbone were synthesized and characterized in our previous publication.^[^ ^26^ ^]^ Single gas transport properties of the membrane studied previously^[^ ^26^ ^]^ are shown in **Figure** **4** and **Table** **2**.

**Table 2:** Single gas permeability, permeance, and idea selectivity in the dense and TFC block co‐polyimide 6FDA‐Durene/6FDA‐CARDO (5000/5000) membranes at 7 bar feed pressure and at 35 °C

Membrane type	Permeability [barrer]				Ideal selectivity		
	N_2_	CH_4_	He	CO_2_	N_2_/CH_4_	He/CH_4_	CO_2_/CH_4_
Dense film	6.53	4.9	160	175	1.34	32.90	36.08

Membrane type	Permeance [GPU]				Ideal selectivity		
	N_2_	CH_4_	He	CO_2_	N_2_/CH_4_	He/CH_4_	CO_2_/CH_4_
Multilayer thin film	2.7	2.1	58.4	72.0	1.29	27.81	34.29

As described in our previous publications,^[^ ^25^, ^26^ ^]^ the substitution of the CARDO diamine containing fluorenyl moieties in the backbone of the polyimide, instead of mPDA (1,3‐phenylenediamine), exhibited an enhanced performance as the improved performance of this material slightly exceeded the 1991 Robeson upper bound plot, as shown in Figure 4. This enhanced separation performances displayed by the membrane are very competitive and even much better than the performance exhibited by some of the high performance membranes reported in the literature (Figure 4). It should be noted that the proposed revised new 2019 upper bound plot^[^ ^61^ ^]^ is also depicted in Figure 4.

### 3.3 Single and Mixed Gas Permeation Properties of Multilayer Block Co‐Polyimide Thin Film Composite Membrane

The single gas transport properties of the TFC using PTMSP as the gutter layer material is shown in Table 2. The ideal selectivity of CO_2_ over CH_4_ is around 34.2, which is slightly lower than the value of around 36 obtained in the dense layer self‐standing membrane. Similarly, He/CH_4_ ideal selectivity is around 27.8, which is also slightly lower than the value of 33 obtained in the dense membrane. This behavior could be attributed to the fact that the transport behavior of the TFC membranes are usually different from those of the self‐supporting dense membrane. This may be attributed to the different polymer chain arrangements in TFC and dense films,^[^ ^1^, ^2^, ^3^, ^4^ ^]^ which then result to different permeation properties and separation performances.^[^ ^12^ ^]^

The membrane performance was also characterized under sweet mixed gas conditions at around 25 °C, upstream pressure of up to 55 bars, and a stage‐cut of up to 20% with gas mixtures containing 10, 59, 1, and 30 vol% CO_2_, CH_4_, C_2_H_6_, and N_2_, respectively (i.e., no H_2_S in this mixture), using a constant pressure system.

**Figure 5:**
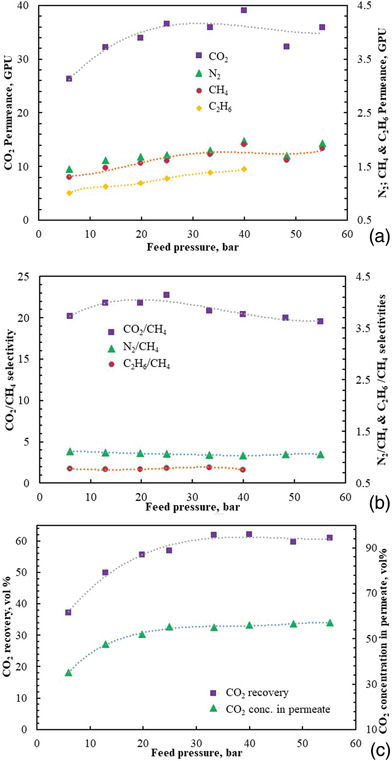
Mixed gases a) permeance, b) selectivity, and c) CO_2_ recovery and concentration in permeate in the multilayer thin film block co‐polyimide 6FDA‐Durene/6FDA‐CARDO (5000/5000) composite membrane versus feed pressure at 25 °C and 5% stage‐cut using mixed gas containing 10, 59, 1, and 30 vol% CO_2_, CH_4_, C_2_H_6_, and N_2_, respectively.

As depicted in **Figure** **5**, mixed gas permeance of all gases initially increase as the pressure increases up to 28 bar, and then somewhat remain constant (reached plateau) as pressure increases further to 55 bar. Furthermore, selectivities of all gases with respect to methane remain somewhat constant as the pressure increases (Figure 5b), indicating the membrane is strong enough to withstand the effect of mixed gas and high pressure as an insignificant plasticization effect could be observed up to 55 bar. The stage‐cut was maintained at 5%, while the feed pressure was increased in this study. As previously reported,^[^ ^43^ ^]^ the competitive sorption of components is usually dominant at low pressure, which leads to a decline in permeance. However, as the pressure increases, the driving force for mass transfer increases due to the higher chemical potential difference across the membrane. Thus, the permeation of slow penetrants that include N_2_, CH_4_, and C_2_H_6_ through the membrane also increases. At a 5% stage‐cut, the partial pressure difference of CO_2_ can approach maximum and the residence time is now used for CO_2_ permeation across the membrane.

Figure 5c shows effect of an increase in pressure on CO_2_ recovery and concentration in the permeate and, as can be observed, both initially increase as the pressure increases up to a value of 28 bar, and then somewhat remain constant (reached plateau) reaching value of 60% and 58%, respectively, as pressure increases further to 55 bar. In order to make the process more economical, CO_2_ recovery should be high enough. Thus, at a stage‐cut of 5% and 6 bar, about 37% of the CO_2_ was recovered. However, this value sharply increased to more than 60% at 55 bar.

**Figure 6:**
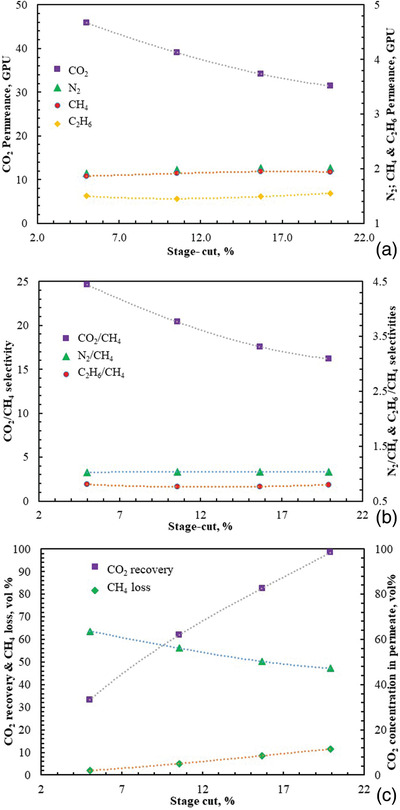
Mixed gases a) permeance, b) selectivity, and c) CO_2_ recovery and concentration in permeate in the multilayer thin film block co‐polyimide 6FDA‐Durene/6FDA‐CARDO (5000/5000) composite membrane versus stage‐cut at 41.4 bar and 25 °C using mixed gas containing 10, 59, 1, and 30 vol% CO_2_, CH_4_, C_2_H_6_, and N_2_, respectively.

**Figure** **6** shows the influence of an increasing stage‐cut on mixed gas permeation properties. Both CO_2_ permeance and CO_2_/CH_4_ selectivity in the mixed gas decline as stage‐cut increases. However, N_2_, CH_4_, and C_2_H_6_ permeances and N_2_/CH_4_ and C_2_H_6_ selectivities somewhat remain constant with an increasing stage‐cut, as shown in Figure 6a,b. The decrease in CO_2_ permeance and CO_2_/CH_4_ selectivity with an increase in stage‐cut is as a result of decline in CO_2_ concentration in the permeate and an increase in CH_4_ loss as the stage‐cut is increased, as can be seen in Figure 6c. Also observed in Figure 6c is the decrease in retentate CO_2_ as the stage‐cut increases, leading to an increase in the CO_2_ recovery rate. It should be noted that the decrease in CO_2_ purity in the permeate is attributed to the fact that there is insufficient CO_2_ in the feed to withstand a high permeate CO_2_ concentration. This is a case of the typical recovery/purity trade off usually seen in most membrane processes, and similar behavior was observed in a previous publication.^[^ ^11^, ^54^ ^]^

### 3.4 Sour Gas Mixture Transport Properties of Multilayer Block Co‐Polyimide Thin Film Composite Membrane

One of the aims of gas separation membrane development is to find an application in sour gas separation from natural gas reserves. Thus, in an attempt to mimic aggressive environments, the permeation properties of the 6FDA‐Durene/6FDA‐CARDO (5000/5000) TFC membrane was studied using a sour mixed gas containing 10, 10, 59, 1.0, and 20 vol% of H_2_S, CO_2_, CH_4_, C_2_H_6_, and N_2_, respectively. Permeance of all the penetrants that include H_2_S, CO_2_, CH_4_, C_2_H_6_, and N_2_ and CO_2_/CH_4_ and H_2_S/CH_4_ selectivities are shown in **Table** **3** for the sour gas mixture with H_2_S composition of up to 10 vol% and at an upstream pressure of up to 48 bar. The CO_2_/CH_4_ and H_2_S/CH_4_ selectivities range from 8.4 to 10.3 and 15.2 to 19.3, respectively, while CO_2_ and H_2_S permeabilities are up to 122 and 220 GPU, respectively. This separation performances displayed by the membrane are very competitive in comparison to the performance exhibited by some of the high performance membranes reported in the literature,^[^ ^41^ ^]^ keeping in mind that the available reported data represent some variation on the upstream pressure (up to 34.5 bar) and the sour gas mixtures used such as the H_2_S‐content (5 to 20 vol%) and the absence of N_2_ and heavier hydrocarbon C_2_H_6_. For instance, Babu^[^ ^41^ ^]^ reported high performance cross‐linkable TEGMC asymmetric hollow fiber membranes, which showed CO_2_/CH_4_ and H_2_S/CH_4_ selectivities of 55 and 29, respectively, for a 5% H_2_S, 45% CO_2_, and 50% CH_4_, and for 20% H_2_S, 20% CO_2_, 60% CH_4_ mixed gas feed with 34.5 bar feed pressure, the CO_2_/CH_4_ and H_2_S/CH_4_ selectivity values were 47 and 22, respectively. Even though these selectivity values reported may be higher than those obtained in this study, however, all the penetrants permeance obtained in this study are an order of magnitude higher than those obtained in the Babu study.^[^ ^41^ ^]^

**Table 3:** Sour gas mixture permeance and selectivity coefficients in the block co‐polyimide 6FDA‐Durene/6FDA‐CARDO (5000/5000) TFC membrane versus feed pressure at 25 °C (gas mixture composition: 10% H_2_S, including 10% CO_2_, 59% CH_4_, 20% N_2_, and 1% C_2_H_6_)

H_2_S comp. [%]	Feed pressure [psi]	Permeance [GPU]					Selectivity	
		N_2_	CH_4_	C_2_H_6_	CO_2_	H_2_S	CO_2_/CH_4_	H_2_S/CH_4_
10	350	6.5	11.6	18.0	120	223	10.3	19.3
	700	7.7	14.5	24.0	122	220	8.40	15.2

## 4 Conclusion

This study presents the development of a multilayer TFC membrane from the block co‐polyimide containing a 6FDA‐Durene/6FDA‐CARDO (5000:5000) backbone, poly[1‐(trimethylsilyl)‐1‐propyne] (PTMSP) and mesoporous polyacrylonitrile (PAN) as selective, gutter, and support layers, respectively. A thin layer of the co‐polyimide selective layer was successfully deposited on a porous PAN support containing a PTMSP gutter layer to form a defect‐free TFC membrane. The co‐polyimide employed as the selective layer in this study was previously developed. Thus, mixed gas transport properties of the TFC membranes were analyzed for N_2_, CH_4_, C_2_H_6_, and CO_2_ gases at a feed pressure of up to 55 bar. The sour gas mixture separation properties were also measured at 25 °C and feed pressure of 48 bar using a sour gas containing 10, 10, 59, 1.0, and 20 vol% of H_2_S, CO_2_, CH_4_, C_2_H_6_, and N_2_ respectively. The TFC membrane exhibited good pure CO_2_ and He permeances of 72 and 58 GPU, respectively, and very good CO_2_/CH_4_ and He/CH_4_ ideal selectivities of 34 and 28 respectively were obtained. These values are comparable to the CO_2_/CH_4_ and He/CH_4_ ideal selectivities of 36 and 33, respectively, obtained for the corresponding dense membranes. The TFC membrane also showed good performance under high H_2_S environment (i.e., H_2_S content of up to 10 vol% H_2_S and feed gas pressure of up to 48 bar). The membrane exhibited CO_2_/CH_4_ and H_2_S/CH_4_ selectivities ranges from 8 to 10 and 15 to 19, respectively, and CO_2_ and H_2_S permeances were up to 122 and 220 GPU, respectively. These separation performances displayed by the membrane are very competitive and even much better than the performance exhibited by some of the high performance membranes reported in the literature. To our knowledge, the feed conditions of sour gas applied in this study are, one of the most aggressive environments applied on TFC membrane in the literature.

## Conflict of Interest

The authors declare no conflict of interest.
